# Lessons from a National Liquid Biopsy Program to Provide Cancer Testing and Treatment for Patients with Advanced Solid Tumors †

**DOI:** 10.3390/curroncol33010018

**Published:** 2025-12-29

**Authors:** Anna Lapuk, Benjamin L. S. Furman, Pedro Feijao, Ebru Baran, Sonal Brahmbhatt, Betty Chan, Ka Mun Nip, Adrian Kense, Brenda Murphy, Ruth Miller, Vincent Funari, Alicja Parker, Melissa K. McConechy, Shaqil Kassam, Arif A. Awan, Bryan Lo, Daniel Breadner, Barry D. Stein, David G. Huntsman

**Affiliations:** 1 Avitia Inc., Montreal, QC H2S 3H1, Canada; 2 Imagia Canexia Health, Vancouver, BC V6R 1P2, Canada; 3 Stronach Regional Cancer Centre, Southlake Regional Health Centre, Newmarket, ON L3Y 2P9, Canada; 4 Ottawa Hospital Research Institute, Ottawa, ON K1Y 1J8, Canada; 5 Division of Medical Oncology, Department of Medicine, The Ottawa Hospital, Ottawa, ON K1H 8M5, Canada; 6 Department of Anatomical Pathology, The Ottawa Hospital, Ottawa, ON K1H 7W9, Canada; 7 Verspeeten Family Cancer Centre, London Health Sciences Center, London, ON N6A 5W9, Canada; 8 Colorectal Cancer Canada, Montreal, QC H3Z 2P9, Canada

**Keywords:** liquid biopsy, NGS, biomarkers, precision oncology, health economics

## Abstract

Personalized cancer treatment depends on the accurate and timely detection of the patient tumor variants. LBx enables minimally invasive tumor mutation profiling. We report results of a pan-Canadian LBx program for patients with advanced solid tumors. Plasma samples were tested at Imagia Canexia Health accredited laboratory using the clinically validated Follow It 38-gene panel. A proprietary platform was used to identify clinically relevant variants in the circulating tumor DNA and report results following accepted international guidelines on clinical significance. A total of 4229 eligible patients submitted samples for LBx testing, and reports for 97% of them were delivered within ~8 days. More than 80% of Canadian oncologists from >150 institutions across 12 provinces (11% from rural centers) participated in the project. The patient cohort consisted mostly of advanced or metastatic lung, breast, and colon cancers. ctDNA mutations were detected in >50% of cases, and clinical trials were recommended for 76% of all participants. Health economics modeling analysis found that Follow It^®^ in combination with tissue biopsy was cost-saving and resulted in an additional 0.1138 QALYs gained relative to tissue biopsy alone. The successful pan-Canadian implementation of a cost-effective, robust LBx testing program demonstrated its sustained demand and feasibility, and its potential economic and health benefits.

## Simple Summary

Detection of the patient-specific mutations present in an individual tumor is critical for the selection of the best treatment option for cancer patients. Liquid biopsy (LBx) allows the detection of such mutations in a less invasive manner and is often faster than traditional tissue biopsy. Here we report a successful experience of running an LBx program for Canadian patients with advanced solid tumors. The testing was done over the course of three years for >4000 patients referred from >150 institutions. A total of 97% of patients received high-quality testing results within an average 8 days, which provided oncologists with actionable information for treatment selection. This study has demonstrated the feasibility and growing demand for LBx testing in Canada with the potential to improve patient outcomes, while allowing the healthcare system to operate more efficiently.

## 1. Introduction

Cancer remains the leading cause of death in Canada, with lung, breast, and colorectal cancers being among the most common newly diagnosed cancers [1]. Targeted therapies available for cancers have shown to improve clinical outcomes [2,3,4,5,6]. The use of these treatments is contingent on relevant biomarker testing, and their tissue-based evaluation is currently the standard of care for many indications. Due to the diversity of oncogenic mechanisms and availability of multiple therapies targeting them, a concurrent evaluation of multiple biomarkers is currently recommended for many solid tumors. For this purpose, the next-generation sequencing (NGS) multi-gene panels have been increasingly recognized as the practical choice [7,8,9,10,11].

Although tissue-based molecular testing has become a mainstay of precision oncology, a number of challenges limit its application. These include availability, lack or insufficiency of tissue material, bone-only disease, difficult-to-biopsy tumor sites, risks associated with tissue collection, and delays in tissue biopsy and molecular testing due to healthcare operational challenges. This is particularly important in Canada, where the level of readiness for genomic-based testing is still low in many provinces, and wait times for surgeries and biopsies are long [12]. This makes the timely reporting of biopsy test results difficult and can increase the average turnaround time between tissue sampling and initiation of treatment to several weeks; it also can lead to suboptimal therapy selection in the absence of molecular information [13,14]. Delays in tissue biopsies have been associated with 17% of patients dying or becoming unsuitable for treatment, and 49.7% not receiving targeted therapies due to delays in biomarker test results [15,16].

Liquid biopsy (LBx) has emerged as an accurate, efficient, and cost-effective tool allowing for minimally invasive tumor mutation profiling. LBx assays can detect mutations in patients’ plasma containing free circulating tumor DNA (ctDNA) and RNA (ctRNA) shed by solid tumors. This information is valuable for therapy selection, as well as monitoring of treatment response, resistance, and disease progression, especially in scenarios when tissue material is limited, unavailable, difficult to obtain safely, or delayed [17]. In Canadian real-world setting, LBx has demonstrated the ability to reduce diagnostic delays and improve access to precision cancer care [18,19,20,21,22,23]. Additionally, LBx can overcome the tissue limitations related to spatial and temporal heterogeneity, providing a more accurate mutational snapshot of a patient’s disease [24]. Despite these benefits and emerging LBx testing recommendations [9], the use of liquid biopsy is still limited across Canada for several reasons, including lack of reimbursement, lack of local molecular testing infrastructure and expertise, and lack of cost-effective, in-house solutions with proven performance.

In response to these challenges, a pilot project named ACTT (***A***ccess to ***C***ancer ***T***esting & ***T***reatment), to bring ctDNA testing into the Canadian healthcare system was launched in July 2020, reaching more than 4000 advanced cancer patients over 3 years, who were tested with the LBx assay Follow It^®^( Imagia Canexia Health, Vancouver, BC, Canada). Follow It^®^ is a high-quality, robust, plasma-based NGS assay developed and rigorously tested for clinical use following AMP/CAP guidelines [25,26]. Follow It^®^ targets actionable genomic mutations in 38 cancer-associated genes, including single-nucleotide variants (SNVs), small insertions and deletions (indels), copy number variants (CNVs), and microsatellite instability (MSI), providing treatment guidance. Designed as a pan-cancer solution for in-house use, Follow It^®^ detects clinically actionable genomic mutations in adult patients with advanced solid cancers, such as lung, breast, and colorectal. This report summarizes the experience of running Follow It^®^ LBx testing in a real-world pan-Canadian setting and provides an early evaluation of its health benefits and cost-effectiveness based on a representative subset of advanced lung cancer patients in a single province.

## 2. Materials and Methods

Blood was drawn in two 10 mL Streck^TM^ DNA BCTs (Streck, La Vista, NE, USA) and sent to the CAP/CLIA/DAP accredited Imagia Canexia Health laboratory for testing using the clinically validated Follow It^®^ liquid biopsy assay. Plasma was isolated using a double spin protocol, where blood was centrifuged at 1600× *g* for 15 min at room temperature, and the upper phase was collected and subsequently centrifuged at 3000× *g* for 10 min at room temperature. Plasma cell-free DNA (cfDNA) was extracted using an optimized Promega Maxwell RSC method, its quality assessed with the Agilent Bioanalyzer High Sensitivity DNA Kit (Agilent Technologies, Santa Clara, CA, USA), and it was quantified using the Qubit™ dsDNA HS Assay Kit (Thermo Fisher Scientific, Waltham, MA, USA) (Supplementary Table S1). Extracted cfDNA was amplified using the multiplex amplicon-based hotspot 30- (v4) or 38-gene (v5) panel and sequenced on Illumina sequencer (Supplementary Table S2A). An in-house developed bioinformatics pipeline and reporting platform were used to identify pathogenic single nucleotide variants (SNVs), including splice site mutations associated with splicing, indels (insertions and deletions), as well as amplification in 9 genes as part of the v5 assay (Supplementary Table S2A–C, Supplementary Figures S1 and S2). The platform has been clinically validated according to CAP/AMP guidelines for NGS assays [25]. Stringent quality criteria and established thresholds for coverage, probability, and tier level of clinical significance were applied in the mutation calling pipeline. The lower limit of detection for SNVs and indels was 0.5% and 0.375% VAF for v4 and v5, respectively, and CNVs were reported based on the strength of evidence provided by the CNV detection pipeline (amplifications or likely amplifications). All indels, complex events, and SNV variants with VAF close to reportable thresholds were manually inspected and validated using the Integrative Genomics Viewer (IGV v2.18.2) [27].

Data visualization was performed using maftools [28]. Pairwise gene–gene mutation interactions were evaluated using the somaticInteractions function from maftools, which applies Fisher’s exact test to mutation matrices to identify significant mutual exclusivity or co-occurrence patterns. Reported p-values are adjusted for multiple testing using the Benjamini–Hochberg false discovery rate (FDR) procedure.

For the cost and health benefit analysis that was focused on non-small cell lung cancer (NSCLC), the model structure was developed, which consists of a decision tree to describe the testing pathway and a Markov model to follow up patients over time (Supplementary Figures S3–S5). The model was parameterized from secondary sources as a result of extensive evidence synthesis activities, and applied for patients in the standard care arm (tissue biopsy alone) and those in a Follow It^®^ arm (tissue biopsy plus Follow It^®^). Standard of care recommendations regarding tissue biomarker testing that existed at the time of this study were used in the modeling (Table 1) [29]. The primary outcome measure was the quality-adjusted life year (QALY), where one QALY is defined as one year lived in perfect health. The model parameterization and assumptions included NSCLC biomarkers used in standard of care or in Follow It^®^ testing, treatment regimens, cost, and accuracy of tissue biopsy and Follow It^®^. Only mutations with a clear pathway to targeted therapy and its associated improved patient outcomes were considered (Table 1), with the presence of these mutations in a patient being mutually exclusive [29]. It was also assumed that the identification of any of the included mutations would lead to a patient being offered targeted therapy. Simplified treatment regimens for NSCLC considered for the current modeling are summarized in Table 2, and the costs were informed by the best available secondary sources. Other parameters, retrieved from published literature, included patients’ clinical outcomes on included treatments, feasibility and delays of tissue biopsy (Supplementary Tables S6–S12). The performance of the Follow It^®^ test used in the analysis (sensitivity 100% and specificity 99%) was based on the prior clinical validation of the assay under CAP/CLIA guidelines (data on file), and the testing cost used in the modelling was $1200 CAD, which included institutional overhead.

## 3. Results

### 3.1. Nationwide Liquid Biopsy Testing Experience

Canada, by area, is the second-largest country in the world, with population centers scattered across the entire country. Due to the critical importance of timely diagnosis, testing would ideally be done in a local or regional facility. However, due to the condensed time frame of the project ACTT, liquid biopsy testing was performed centrally at Imagia Canexia Health’s CLIA, CAP, and DAP-accredited laboratory in Vancouver, British Columbia. Project collaborators, including Lifelabs, Genolife, Ichor Blood Services, Eastern Ontario Regional Laboratory Association, and hospital systems across Canada, drew blood at the local facilities and shipped blood samples to Imagia Canexia Health for testing. Samples were tested using Follow It^®^ assay [26] and analyzed and reported using a proprietary bioinformatics and clinical reporting platform. Reports covering mutation detection results and their interpretation were issued to ordering physicians via fax. The vast majority of blood specimens were received within 7 days of collection, with ctDNA yield ranging from 0.13 to 246 ng/uL, and a mean of 2.10 ng/uL (±7.07 ng/uL). The yields were similar across all three major cancer types (lung, breast, and colon) with colon samples showing slightly higher average ctDNA concentrations, consistent with published observations [30,31] (Supplementary Table S1).

**Figure 1:**
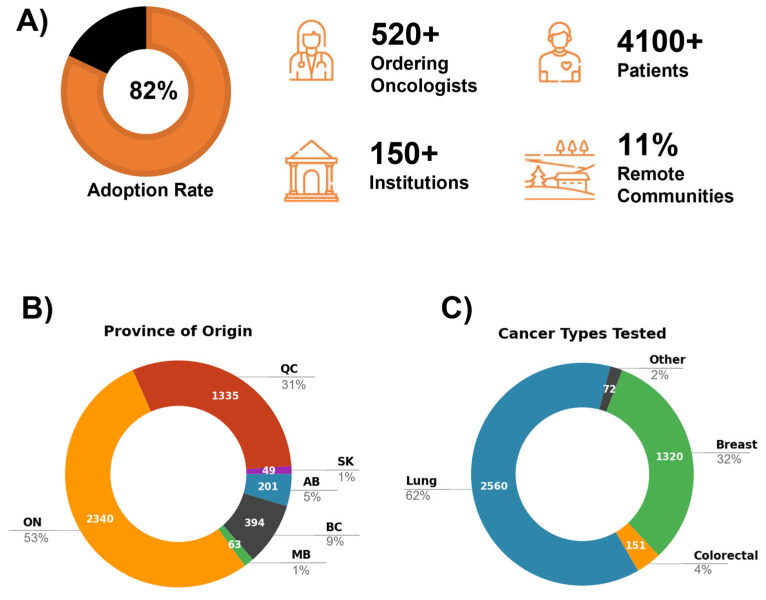
Project ACTT statistics. (**A**) Liquid biopsy usage across Canadian institutions; the adoption rate reflects the fraction of all medical oncologists registered in Canada at the time of the study (n = 642) [32] who used the Follow It^®^ test (n = 527). (**B**) Samples tested per province. (**C**) Cancer types tested.

From July 2020 to August 2023, a total of 4229 eligible patients submitted their samples for liquid biopsy testing using Follow It^®^ assay. For 4103 of those, ctDNA was isolated, and a successful clinical report was produced, indicating an overall testing success rate of 97%. The median turnaround time (TAT) from sample receipt to report send out was 8 days (3–32 days). Oncologists overwhelmingly responded positively to the pilot project, with 82% of all registered oncologists working across 150 institutions ordering the test. The project reached patients in 12 provinces and territories, with the highest participation in Ontario, Quebec, and British Columbia (Figure 1). In the province of Ontario (ON), the main participating centers included Ottawa Hospital, London Health Sciences Center, Niagara Health, Stronach Regional Cancer Centre, Windsor Regional Cancer Program, and Markham Stouffville Hospital.

In the province of Quebec (QC), the University of Montreal Hospital Centre (CHUM), Jewish General Hospital, and University Institute of Cardiology and Respirology of Quebec (IUCPQ) were among the top users. British Columbia (BC) repeated users included BC Cancer Agency (BCCA), Delta, and Richmond Hospitals. The project also exceeded its target of reaching patients in remote and rural areas, with 11% of samples received from outside of major urban centers. Examples include Thunder Bay Regional Health Sciences Centre, Health Sciences North (Sudbury), and Bluewater Health in ON; Regional Hospital Center of Lanaudiere, Centre Hospitalier Regional de Lanaudiere (CHDL) in QC; and BC Cancer—Prince George and Vernon Jubilee Hospital in BC. Other participating rural centers across Canada included Jack Ady Cancer Centre in AB, Cape Breton Cancer Centre in NS, and PEI Cancer Treatment Centre in PEI.

### 3.2. Patient Cohort and Program Performance

The patient cohort of 4103 samples consisted of advanced or metastatic solid cancers of the lung (2560, 62%), breast (1320, 32%), and colon (151, 4%), with remaining cases coming from a mix of multiple solid tumor types, such as pancreatic, GI, and gynecological cancers (Figure 1). The cohort consisted mostly of stage IV (78%) and III (5%) cancers with a median patient age of 67. All samples were assessed for SNVs and indels, and 2369 samples had additional copy number data for nine genes (v5 panel). Across three major cancer types (breast, lung, colorectal), in ~50% of cases, one or more mutations were identified, reflecting published frequencies of detectable mutations in the plasma of these patients [30,33,34]. In 37% of all samples, at least one tier I/II mutation was detected and targeted treatments were recommended. One or more clinical trials were recommended for 76% of all participants. According to the information provided on sample requisitions, 49% (n = 2053) of patients had no previous molecular testing at the time of liquid biopsy testing. Of those, 54% (n = 1095) were ctDNA mutation-positive, and 36% (n = 729) had one or more tier I/II mutations detected. Altogether, we detected a total of 3695 SNVs and indels across 4103 samples, and 395 CNVs across 2324 samples. Mutation data were summarized across the entire cohort using overlapping content of v4 and v5 panels, excluding SNVs/indels within an additional eight genes unique to v5 (total of 90 mutations from *DICER1*, *FGFR1-3*, *FOXL2*, *NTRK1*/*3*, and *STK11*; data available in Supplementary Table S2B and Supplementary Figure S1).

### 3.3. Lung Cancer Cohort

**Figure 2:**
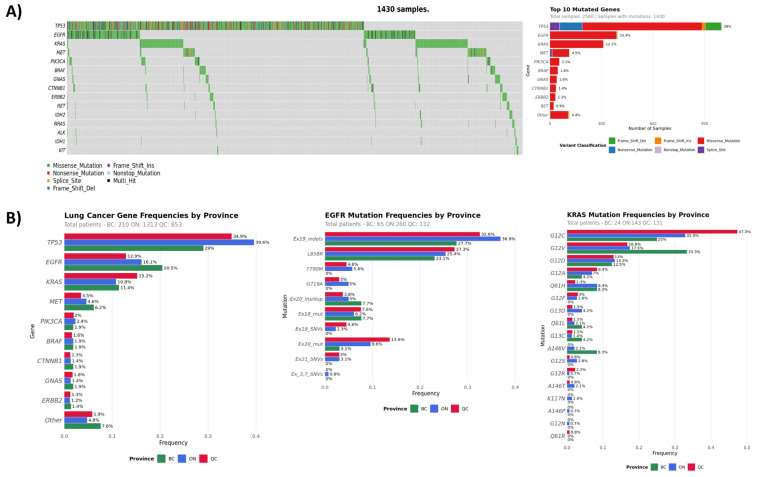
Lung cancer cohort mutation spectrum. (**A**) Oncoplot of genes with mutations. (**B**) Gene and mutation relative frequencies by province.

The lung cancer cohort consisted predominantly of NSCLC (90%). Among tested plasma samples (n = 2560), 56% harbored pathogenic mutations in ctDNA, with the most commonly mutated genes being *TP53* (36%), *EGFR* (15%), *KRAS* (12%), *MET* (4%), *PIK3CA* (2%), and *BRAF* (2%) (Figure 2, Table 3). Tier I/II mutations were detected in 38% of all lung cancer patients. In patients with no previous molecular testing, liquid biopsy detected a total of 640 tier I/II mutations in 543 patients, with top mutated genes being *EGFR*, *KRAS*, *MET*, and *BRAF*. For those patients who had previous molecular testing, we detected additional ctDNA mutations in key biomarkers. These included *EGFR* and *KRAS* mutations in 11% (47/409) and 26% (107/409) of patients, respectively. Interestingly, out of 47 samples with *EGFR* ctDNA mutations, 14 cases had previously been reported to be PD-L1-positive. PD-L1 expression is an indication for immune-checkpoint inhibitor (ICI) therapy. ICI is less effective among patients harboring driver mutations, such as *EGFR*, with some evidence showing increased risk of adverse effects [35]. Our findings indicate the importance of timely multi-gene testing for optimal therapy selection.

The most frequently detected *EGFR* mutations included exon 19 indels (25.3%) and *EGFR*-L585R (32.7%) (Supplementary Figure S2). These commonly observed oncogenic mutations are associated with response to EGFR tyrosine kinase inhibitor (TKI) treatment [36,37,38,39]. T790M resistance mutation relative frequency was 5.6%. Osimertinib is approved by the FDA and Health Canada for the treatment of advanced NSCLC with the T790M mutation that progressed on prior EGFR TKI, and for first-line treatment of *EGFR* mutation-positive NSCLC (L858R and exon 19 deletions). *KRAS*-G12C (37.6%) and *KRAS*-G12V (18.6%) were the most commonly observed *KRAS* mutations (Supplementary Figure S2). NSCLC harboring *KRAS*-G12C mutations have been predicted to respond to sotorasib therapy and have FDA and Health Canada approval [8].

While the overall mutational spectrum in the lung cancer cohort was similar to published data [34], we observed some inter-provincial differences. BC patients showed the highest prevalence of *EGFR* mutations (21%) followed by *KRAS* (11.4%), whereas in ON and QC patients, *KRAS* mutations were observed more frequently relative to *EGFR* (Table 4, Figure 2). *KRAS* mutations, particularly G12C, are known to be smoking-related [40,41]. Our observation of relative *EGFR* and *KRAS* mutation frequency differences, although marginally statistically significant due to low numbers, correlates with the smoking rates in respective provinces, with BC having the lowest and QC the highest of the three [42].

Gene amplification was assessed in 1870 lung samples, with ~12% of cases showing copy number gains in one of the nine genes. *MET* was the most frequently amplified (3.6% combined CNVs, Table 5). *MET* amplifications are present in approximately 1–5% of all NSCLC patients [43,44]. It is a common resistance mechanism to EGFR tyrosine kinase inhibitor (TKI) therapy in NSCLC with or without *EGFR* mutations, resulting in activation of downstream pathways, and is associated with a poor prognosis. Crizotinib, capmatinib, and tepotinib are suggested for the treatment of patients with high-level *MET*-amplified NSCLC [45] or *MET* exon 14 skipping, and other new combinations or novel therapeutic agents are currently under investigation [2,43].

### 3.4. Breast Cancer Cohort

**Figure 3:**
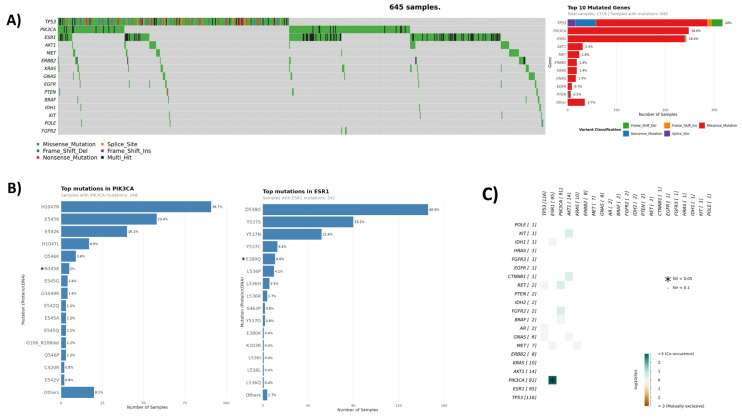
Breast cancer cohort mutation spectrum. (**A**) Oncoplot of genes with ctDNA mutations. (**B**) Mutation relative frequencies in select genes. *: *PIK3CA*-N345K and *ESR1*-E380Q frequencies are shown relative to the overall breast cancer cohort. Since these hotspots were introduced in the v5 assay, their corrected frequencies are 5.1% and 10.8% respectively (data shown in Supplementary Figure S1). (**C**) Co-occurrence plot of genes with ctDNA mutations.

The breast cancer cohort consisted of 1320 samples, 644 (49%) of which harbored pathogenic ctDNA mutations, with the most commonly mutated genes being *TP53* (23%), *PIK3CA* (19%), *ESR1* (18%), and *AKT1/MET* (4%). (Figure 3, Table 3). Tier I/II mutations were detected in 481 (36%) of all breast cancer samples, the majority of which (279/58%) having ≥1 mutation in the PI3K/AKT pathway (*PIK3CA*, *AKT1*, and *PTEN*) (20% total cohort frequency). The most frequent mutations in *PIK3CA* included exon 20 mutation kinase domain H1047R and exon 9 helical domain mutations E545K/E542K (Figure 3). In the *ESR1* gene, frequently observed mutations were D538G, Y537S or Y537N or Y537C, and E380Q. Interestingly, 30% of samples with mutated *ESR1* (73/243) harbored ≥2 concurrent *ESR1* ctDNA mutations. *ESR1* mutations are associated with acquired resistance to antiestrogen therapies, and multiple *ESR1* mutations possibly indicate resistance subclones.

In patients with no previous molecular testing (n = 436), LBx detected a total of 232 tier I/II mutations in 156 samples with top mutated genes being *ESR1*, *PIK3CA*, *AKT1*, and *ERBB2*, providing actionable information in the absence of other molecular testing. In 57% of cases with available previous molecular testing information (508/884), the records indicated the cancer subtype of hormone receptor (ER, PR)-positive with/without HER2-negative status. In this specific subgroup, 49% harbored ctDNA mutations, the majority of which (61%) were *PIK3CA* and/or *ESR1* mutations (Table 6). Mutation interaction analysis identified a significant co-occurrence of *PIK3CA* and *ESR1* mutations (adjusted *p*-value < 4.02 × 10^−5^) that was observed in 6.9% of HR+ samples (35/508, Figure 3), which is similar to published incidences [46]. PI3K/AKT pathway alterations occur frequently in patients with advanced HR-positive breast cancer, collectively affecting up to 50% [47]. Therapies targeting this patient population, such as capivasertib, inavolisib, and alpelisib, are currently approved by FDA and Health Canada, respectively [48,49,50]. *ESR1* mutations are a common mechanism of resistance of ER+ breast cancer to endocrine therapies like aromatase inhibitors and tamoxifen. These cancers may retain sensitivity to selective estrogen receptor degraders (SERDs), including FDA-approved elacestrant [51] and imlunestrant [52]. Other SERDs are currently being investigated in clinical trials [53], with LBx-based *ESR1* testing serving as a valuable tool to guide treatment [54].

Gene amplification was assessed for 446 breast cancer samples, with ~20% of cases showing copy number gains in one of the nine genes, with *FGFR1* being the most frequent alteration (Table 5). *FGFR1* amplifications are present in approximately 8.47% of breast carcinoma patients [44] and predict endocrine treatment resistance in HR-positive breast cancer. However, these patients may be sensitive to mTOR inhibitors [55] and are currently under clinical investigation with FGFR inhibitors [56,57,58].

The detection rate of *ERBB2* amplification in our breast cancer cohort was low compared to tissue-based rates, which are approximately 15–20%. This can be attributed to a bias in our breast cancer cohort composition and fundamental limitations in CNV detection from cfDNA using small targeted panels. Our breast cancer cohort consisted of only 2.5% of cases with unequivocal HER2-positive status on tissue as indicated by the previous molecular testing. Although the study was open to all advanced cancer patients, such bias can be explained by oncologists’ preferences for obtaining LBx testing for their patients, rendering the breast cancer cohort not fully representative of the advanced breast cancer patient population. Further, it is well established that CNV detection requires a higher tumor fraction and greater genomic coverage than point mutation detection, leading to generally lower sensitivity of LBx assays [59,60,61]. Although our findings underscore a known technical limitation of LBx for CNV detection, we note that the rate of CNV calls in our study was still higher than in a recent comparable study by Nicholas et al. using a similar small NGS panel [23]. Finally, LBx-based *HER2* amplification detection in advanced breast cancer patients is challenged by the fact that the primary tissue status may not necessarily reflect that of metastatic disease throughout the body, leading to tissue-plasma discordance [62]. For example, Abraham et al. reported only 30% sensitivity of *HER2* amplification detection using Guardant360^TM^ LBx assay in metastatic breast cancer patients with confirmed HER2-positive status in tissue [63].

### 3.5. Colon Cancer Cohort

**Figure 4:**
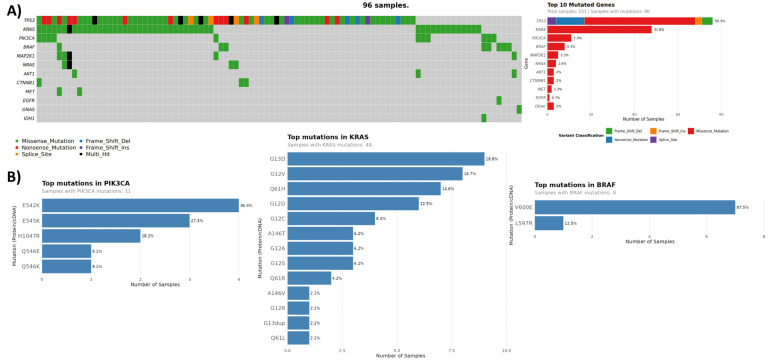
Colon cancer cohort mutation spectrum. (**A**) Oncoplot of genes with ctDNA mutations. (**B**) Mutation relative frequencies in select genes.

The colon cancer cohort consisted of 151 samples, 96 (64%) of which harbored pathogenic mutations in their ctDNA, with the most commonly mutated genes being *TP53* (50%), *KRAS* (32%), *PIK3CA* (7%), and *BRAF* (5%) (Figure 4, Table 3). Tier I/II mutations were detected in 40% of all colorectal cancer patients. In patients with no previous molecular testing, liquid biopsy detected a total of 26 tier I/II mutations in 20 patients, with *KRAS* and *PIK3CA* being the top mutated genes. Most frequently observed mutations in *KRAS* across all colon cancer samples involved G12, G13, and Q61 codons, with a combined prevalence of 29% (44/151) (Figure 4). Consistent with published data [64,65], the most frequently observed *PIK3CA* mutations included those in exon 9 involving the helical domain of the protein (positions E542-Q546), followed by exon 20 mutation H1047R in the kinase domain with a combined prevalence of 7% (11/151). The predominant *BRAF* mutation detected was V600E (in 7/8 samples), with a respective cohort prevalence of 5% (7/151). Collectively, mutations in *KRAS*, *PIK3CA*, and *BRAF*, associated with resistance to first-line anti-EGFR monoclonal antibody treatment of mCRC, were detected in 38% of all tested samples (57/151).

Gene amplification was assessed for 26 colon cancer samples, with ~23% of cases showing amplifications in one of the nine genes and *MET* gain being the most frequent event (Table 5). *MET* amplifications have been shown to contribute to anti-EGFR resistance in colorectal cancer, and de novo amplifications are one of the major mechanisms of acquired resistance [66,67]. Although the overall prevalence of *MET* amplifications in colorectal carcinoma is low (0.46% [44]), it is enriched in ctDNA of patients with anti-EGFR refractory disease [68]. These patients may benefit from the use of MET inhibitors, including FDA-approved capmatinib for *MET* exon 14 alterations [67,69].

### 3.6. Cost-Effectiveness and Budget Impact Analysis of Follow It^®^ in NSCLC

For LBx technologies to become equally accessible to all Canadians, public funding is a necessity. To help inform provincial payers about the benefits of Follow It^®^ testing, an economic analysis was conducted in collaboration with the Institute of Health Economics (Edmonton, AB, Canada). We aimed to determine the potential for Follow It^®^ to be cost-effective as an addition to tissue biopsy compared to tissue biopsy alone for patients with advanced NSCLC presenting for genetic tumor profiling within the Ontario health care payer market. The modeling was based on 4350 treatment naïve stage IV or incurable stage III non-squamous NSCLC Ontario patients ≥ 60 years of age, with 50% of patients being male.

The results of the modeling are summarized in Table 7. Follow It^®^ would save an average of $18,569 CAD per patient compared to tissue biopsy alone. In terms of health benefit, Follow It^®^ would provide an additional 0.1138 QALYs gained on average per patient. Given that Follow It^®^ would be more effective and less expensive than tissue biopsy, under the current scenario assumptions, it can be said that concurrent Follow It^®^ and tissue biopsy testing dominates tissue biopsy alone.

The budget impact analysis of implementing Follow It^®^ over a 5-year time horizon indicated that increased annual savings will be made over this period, from $74.2 million CAD in year one, to $84.5 million CAD in year 5 (Supplementary Table S13). The majority of the cost savings are due to savings as a result of treatment selection. Of note, although there are increased costs associated with the targeted therapy, these are more than offset by the total savings associated with the overall benefit of improved diagnostic and therapeutic pathway, including, but not limited to, the optimal therapy selection and therefore reduced rates of adverse effects, reduced delays in time to treatment, and the reduced need for costly combinations of chemotherapy and immunotherapy.

## 4. Discussion

LBx has become an invaluable tool for precision oncology, alleviating some of the challenges associated with tissue testing. This report summarizes the successful implementation of Canada’s first nationwide ctDNA genomic testing program, delivering actionable molecular information within clinically meaningful TAT, and ensuring equitable access to advanced genomic diagnostics for all eligible patients.

The observed high technical success rate of 97% and overwhelming response from the majority of Canadian oncologists across urban and rural centers indicate both the feasibility of establishing liquid biopsy infrastructure on a national scale, and a sustained demand in a rapidly evolving precision oncology space. The assay detected ctDNA mutations in more than half of all patients, with 37% harboring clinically actionable mutations. A total of 76% of patients were matched with clinical trials. Coupled with the clinically meaningful average TAT of 8 days from sample receipt to test results, the project demonstrated the potential to improve patients’ outcomes due to the timely choice of the optimal therapy. Although biomarker testing recommendation is included in NCCN, ASCO, and some of provincial guidelines for breast, colon, and NSCLC, access to molecular testing is still limited across Canadian institutions [70]. In our study, 49% of patients did not have any previous molecular testing. This is in agreement with recent reports on the observed rate of molecular testing of Canadian cancer patients of <30% [14,71]. Additionally, in some cases with limited available information on baseline biomarkers in tissue, Follow it^®^ identified mutations that may interfere with the therapy chosen based on tissue testing alone, such as *EGFR* ctDNA mutations in lung cancer patients with PD-L1 expression. This underscores the value of concurrent liquid biopsy and tissue testing for multiple actionable biomarkers to enable well-informed treatment decisions.

Several other reports focusing on the clinical benefits of Follow It^®^ testing performed within the ACTT project have been published [19,21,72]. In a study published by Desmeules and co-authors (IUCPQ, QC), 91 patients with advanced NSCLC who had previously undergone single gene tumor tissue genotyping for baseline biomarkers were assessed with LBx using Follow It^®^ via the ACTT project [19]. While for this study cohort Follow It^®^ undoubtedly provided additional information relative to tissue testing alone (47% vs. 18%), translation of this information to clinical practice changing treatment was modest (only 5 pts out of 91 changed therapy). This was due to the regulatory challenges at the time of the study, where access to targeted treatments outside of reimbursed indications was limited. Notably, the median TAT of biomarker information availability was shorter for the centralized Follow It^®^ testing compared to the local tissue testing (10 vs. 13 days). Recently, Breadner and colleagues (Verspeeten Family Cancer Centre, London Health Sciences Centre) reported their experience using ACTT provided Follow It^®^ ctDNA testing as part of the standard work-up for patients with advanced NSCLC. The authors demonstrated significant reduction in (i) time to molecular results (14 vs. 35 days), (ii) time from first respirology/thoracic surgery consult to molecular results (22 vs. 48 days), and (iii) time from medical oncology consultation to initiation of first-line treatment (12 vs. 22 days) [72].

The pilot study of 20 patients of never or light smokers with suspected advanced lung cancer, published by Leighl and colleagues (Princess Margaret Cancer Center, University Health Network), reported the use of Follow It^®^ as part of the pre-diagnostic work-up [21]. Compared to the reference cohorts, the mean TAT for biomarker results and time to treatment initiation (TTT) were significantly shorter for the liquid biopsy arm (17.8 vs. 23.6 days, and 32.6 vs. 62.2 days, respectively). Interestingly, the time to treatment was shortened for all patients participating in the plasma-first approach, even if targetable alterations were not identified. With the observed concordance of 71% between plasma and tissue testing results and clear time savings, the authors concluded that a plasma-first approach can increase detection of therapeutically targetable mutations, especially when tissue DNA is insufficient or unavailable, potentially leading to better patient outcomes.

Demonstrating clinically meaningful outcomes for patients, such as quality-of-life gains and cost-effectiveness, is a critical stepping stone to wide adoption and reimbursement of LBx. Using a model-based evaluation, we assessed the clinical and economic benefit of Follow It^®^ as a clinical-grade, next-generation sequencing targeted panel for somatic tumor testing to predict therapy in advanced NSCLC patients. Considered as an addition to tissue biopsy, and compared to the standard care of tissue biopsy alone, Follow It^®^ was found to be cost-effective, saving approximately $18,569 CAD per patient, and resulted in an additional 0.1138 QALYs gained. Based on the test price of $1200 CAD and its demonstrated accuracy, the assay had the potential to provide significant savings to the healthcare system. Of note, Follow It^®^ testing was performed centrally for this study, and further cost savings can be realized if it is run locally [73,74].

Our study had a number of limitations. Although the Follow It^®^ panel covers most of the actionable biomarkers for major solid tumors, it does not detect fusion events that, although relatively rare, are critical for decision-making in lung and other cancers. Also, the CNV detection using LBx is commonly recognized as a methodological challenge. In our study, the overall rate of CNV detection was higher than that reported in other real-world liquid biopsy studies [23,75], and the spectrum of CNV alterations observed in the NSCLC cohort was consistent with established genomic profiles for this cancer. Nevertheless, the compositional bias of our breast cancer cohort was a critical limitation that precluded a comprehensive evaluation of our assay’s performance for *HER2* amplification status, a key biomarker for this malignancy. Further, our cost and health benefit analysis was also constrained by a simplified treatment pathway, an assumption of all biomarker-positive patients receiving targeted treatment, and an evolving landscape of standard-of-care tissue testing practices. Such modeling is recognized to be challenging due to high complexity of actual application of precision oncology principles. Some of the confounding factors include the combination of tests and treatments, patient-level processes and preferences, local vs. centralized testing models, delays in diagnosis and treatment, etc. [74,76]. Institutions operate in a constantly evolving regional regulatory environment and often have inequitable access to diagnostic and therapeutic technologies. Despite all of these limitations, our observations were in agreement with a recently published Canadian report [77] demonstrating similar economic and health benefits of using LBx for NSCLC patients. Together with the growing evidence of the Canadian patients’ preference for less invasive biomarker testing [78], our study highlights the feasibility, need, and benefits of the use of LBx to guide timely treatment decisions and improve outcomes.

We note that our analysis was conducted from the perspective of a Canadian provincial single-payer health system, which necessarily influences both the observed clinical intervention implementation processes and the estimated economic outcomes. Although other jurisdictions may operate under different clinical pathways, reimbursement mechanisms, or pricing structures, the core findings—namely the value of coordinated testing pathways and the potential for cost-efficient adoption of liquid biopsy—remain relevant to health systems pursuing value-based care. Nevertheless, adaptation to local governance, payment models, and data infrastructure would be required for direct translation outside of a single-payer context.

## 5. Conclusions

The need to provide timely diagnostic information for patients and their healthcare providers to select the most effective therapies for their cancer, including the availability of testing closer to home, is increasingly recognized as the priority in Canada. Liquid biopsies have emerged as a promising complement or alternative to tissue biopsy in recent years, which has the potential to provide additional information, reduce turnaround times, decrease suboptimal drug use, and improve treatment selection in oncology, resulting in better outcomes. The Follow It^®^ LBx tool is no exception, with its demonstrated sensitivity and specificity, relatively lower cost, and focus on clinically actionable genomic mutations [26]. Here, we have described the success of running it centrally for Canadian advanced cancer patients, providing high-quality comprehensive information for an informed therapy selection within clinically relevant turnaround times. Local deployment of this test would enable further improvement in overall cost, speed, and accessibility of LBx for patients. The assay has been suggested by the Canada’s Drug Agency (CDA) to have a significant impact on Canada’s healthcare system in the next 1–3 years [79], given its potential to improve patient outcomes and reduce costs. By facilitating timely, cost-effective access to genomic mutation testing, Follow It^®^ is positioned to help Canadian cancer patients receive the best possible care while allowing the healthcare system to operate more efficiently with potential savings.

## Figures and Tables

**Table 1:** Prevalence of oncogenic driver alterations used in the economics analysis [29].

Standard of Care		Follow It^®^	
Mutation	Prevalence	Mutation	Prevalence
*EGFR*	13.5% (12–15%)	*EGFR*	13.5% (12–15%)
*ALK*	5% (2–8%)	*BRAF*	3% (1–5%)
*ROS1*	1.2% (0.7–1.7%)	*MET*	4.5% (4–5%)
		*KRAS*-G12C	12% (9–15%)

**Table 2:** Simplified treatment regimens included in the model of economic benefit. The TKI treatment based on the presence of activating EGFR mutation served as a prototype of a targeted treatment scenario vs. standard chemotherapy.

	EGFR Mutation Detected	No Mutations Identified
First Line	Osimertinib (80 mg, once daily) until disease progression	Pembrolizumab + platinum-based chemotherapy (carboplatin + pemetrexed)
Second Line	Platinum doublet chemotherapy (four 3-week cycles (cisplatin plus pemetrexed) followed by pemetrexed until disease progression	Single-agent chemotherapy (docetaxel)
Third line for disease progression or recurrence	Chemotherapy (docetaxel)	Chemotherapy (pemetrexed)
Further progression	All patients receive best supportive care	

**Table 3:** Most frequently mutated genes in lung, breast, and colon cancer cohorts. NA—not applicable.

Lung		Breast		Colon	
Gene	n (%)	Gene	N (%)	Gene	N (%)
*TP53*	930 (36)	*TP53*	304 (23)	*TP53*	75 (50)
*EGFR*	390 (15)	*PIK3CA*	247 (19)	*KRAS*	48 (32)
*KRAS*	311 (12)	*ESR1*	243 (18)	*PIK3CA*	11 (7)
*MET*	112 (4)	*AKT1*	31 (2)	*BRAF*	8 (5)
*BRAF*	46 (2)	*MET*	24 (2)	*MAP2K1*	5 (3)
*GNAS*	40 (2)	*EGFR*	9 (1)	*AKT1*	3 (2)
*PIK3CA*	55 (2)	*ERBB2*	19 (1)	*CTNNB1*	3 (2)
*CTNNB1*	35 (1)	*GNAS*	17 (1)	*NRAS*	3 (2)
*ERBB2*	32 (1)	Other genes (<=10 each)	62 (5)	Other genes (n <= 3 each)	7 (5)
*IDH2*	16 (1)	no reported variants	676 (51)	no reported variants	55 (36)
*NRAS*	16 (1)	**Total**	**1320 (NA)**	**Total**	**151 (NA)**
*RET*	22 (1)				
Other genes (N <= 10 each)	79 (3)				
no reported variants	1132 (44)				
**Total**	**2560 (NA)**				

**Table 4:** Gene and mutation relative frequency per province in the lung cancer cohort. Gene frequencies are relative to the total number of lung cancer samples tested; mutation frequencies are relative to the total number of respective gene mutations in the lung cancer cohort. * *KRAS*-G12C frequency difference between QC and BC was significant (*p*-value < 0.05, Fisher’s exact test).

Gene	Genes			EGFR Mutations				KRAS Mutations			
	BC	ON	QC	Mut	BC	ON	QC	Mut	BC	ON	QC
*TP53*	30.0%	42.7%	37.3%	Ex19_indels	27.7%	36.9%	32.6%	G12C *	25.0%	32.9%	47.3%
*KRAS*	11.4%	10.8%	15.2%	L858R	23.1%	25.4%	27.3%	G12V	33.3%	17.5%	16.8%
*EGFR*	21.0%	16.2%	12.9%	T790M	0.0%	5.8%	4.5%	G12D	12.5%	13.3%	13.0%
*MET*	6.2%	4.7%	3.5%	G719A	0.0%	5.0%	3.0%	G12A	4.2%	7.0%	8.4%
*PIK3CA*	1.9%	2.4%	2.0%	Ex20_ins/dup	7.7%	5.0%	3.8%	Q61H	8.3%	8.4%	2.3%
*GNAS*	1.9%	1.4%	1.8%	Ex18_mut	7.7%	6.2%	7.6%	G12F	0.0%	2.8%	3.1%
*BRAF*	1.9%	1.9%	1.6%	Ex19_SNVs	0.0%	2.3%	4.5%	G13D	0.0%	4.2%	1.5%
*CTNNB1*	1.9%	1.4%	1.3%	Ex20_mut	3.1%	9.6%	13.6%	Q61L	4.2%	2.1%	1.5%
*ERBB2*	1.4%	1.2%	1.3%	Ex21_SNVs	0.0%	3.1%	3.0%	G13C	4.2%	1.4%	1.5%
*RET*			1.3%	Ex_3,7_SNVs	0.0%	0.8%	0.0%	A146V	8.3%	2.1%	0.0%
*NRAS*		0.7%						Other	0.0%	8.4%	4.6%
*IDH2*	2.9%										
Other	4.8%	4.3%	4.6%								
**TOTAL samples (n)**	**210**	**1313**	**853**		**65**	**260**	**132**		**24**	**143**	**131**

**Table 5:** Gene amplification prevalence per cancer type. CNV calls were categorized based on the strength of evidence as CNV and Putative CNV. Prevalence (%) is shown for CNV subset alone and in combination with Putative CNV.

	LUNG, n = 1870				BREAST, n = 449				COLON, n = 26			
	CNV		Putative CNV		CNV		Putative CNV		CNV		Putative CNV	
Gene	n	%	n	%	n	%	n	%	n	%	n	%
*CCNE1*	2	0.1%	3	0.3%	1	0.2%	0	0.2%				
*EGFR*	26	1.4%	37	3.4%	2	0.4%	2	0.9%				
*ERBB2*	8	0.4%	3	0.6%	3	0.7%	2	1.1%				
*FGFR1*	28	1.5%	11	2.1%	16	3.6%	14	6.7%			1	3.8%
*FGFR2*			55	2.9%	0	0.0%	27	6.0%			3	11.5%
*KIT*	2	0.1%	2	0.2%								
*KRAS*	4	0.2%	1	0.3%	1	0.2%	1	0.4%				
*MET*	31	1.7%	37	3.6%			38	8.5%			5	19.2%
*PIK3CA*	11	0.6%	6	0.9%	4	0.9%	4	1.8%	1	3.8%		3.8%
None	1658	88.7%		88.7%	358	79.7%	0	79.7%	20	76.9%		76.9%

**Table 6:** Gene mutation prevalence in HR+ breast cancers. Prev mol—previous molecular testing.

Samples		n	%
**With Prev Mol Information**		884	
HR+ samples		508	57.5%
**HR+ samples**	w/o ctDNA mut	260	51.2%
	with ctDNA mut	248	48.8%
	with *PIK3CA* mut	92	18.1%
	with *ESR1* mut	95	18.7%
	with *AKT1* mut	14	2.8%
	with *PTEN* mut	2	0.4%
	with *PIK3CA*/*AKT1*/ *PTEN*/*ESR1* mut	165	32.5%

**Table 7:** The benefit of additional Follow It^®^ testing vs. tissue biopsy alone. ICER—incremental cost-effectiveness ratio; costs and QALYs gained are averages per patient.

Scenario	Costs	Incremental Costs	QALYs Gained	Incremental QALYs	ICER
Tissue Biopsy	CAD 225,943		1.4276		
Follow It^®^	CAD 207,374	CAD −18,569	1.5415	0.1138	Dominates

## Data Availability

The original contributions presented in this study are included in the article/Supplementary Material. Further inquiries can be directed to the corresponding author.

## Supplementary Materials

The following supporting information can be downloaded at: https://www.mdpi.com/article/10.3390/curroncol33010018/s1, References [80,81,82,83,84,85,86,87,88,89,90,91,92,93,94,95,96,97,98,99] are cited in the Supplementary Materials. Table S1: ctDNA concentrations. Table S2: (A) Follow It panel content. (B) List of reported SNV and indel mutations. (C) List of reported CNVs. Tables S3–S5: Mutation frequencies per gene and per province for lung, breast, and colorectal cohorts. Tables S6–S12: Input parameters for the health economics analysis. Table S13: Budget impact analysis. Figure S1: *PIK3CA* and *ESR1* mutation relative frequencies in breast cancer by assay version. Figure S2: Relative mutation frequency in the lung cancer cohort for select genes. Figure S3: The model structure for the testing component of standard of care. Figure S4. The model structure for the testing component of the Follow It^®^ arm. Figure S5: Markov models for patients receiving targeted therapy or chemotherapy.
