# Supplementary material for: Lessons from a National Liquid Biopsy Program to Provide Cancer Testing and Treatment for Patients with Advanced Solid Tumors
Source: Curr Oncol. 2025 Dec 29;33(1):18. doi: 10.3390/curroncol33010018 (PMC12840088; doi:10.3390/curroncol33010018)
Supplement: Supplementary file 1 [file curroncol-33-00018-s001.zip › Supplementary Materials.pdf]

Supplementary Materials

Table S1. ctDNA concentrations. (attached as a separate file)

Table S2. A) Follow It panel content. B) List of reported SNV and indel mutations. C) List of reported CNVs. (attached as a separate file)

Tables S3-5. Mutation frequencies per gene and per province for lung, breast and colorectal cohorts. (attached as a separate file)

Figure S1. PIK3CA and ESR1 mutations relative frequencies in breast cancer by assay version. v5 assay upgrade included the addition of a number of rare gene hotspots, that are generally observed at low frequencies in published data compared to prevalent hotspots. Overall, 90 additional SNVs/indels out of total 3695 were detected by v5 assay only (Supplementary table 2B) with only two notable cases of ESR1:E380Q and PIK3CA:N345K in breast cancer cohort. Their frequencies, relative to all gene mutations from across 449 breast cancer samples tested with v5, were 10.8% and 5.1% respectively.

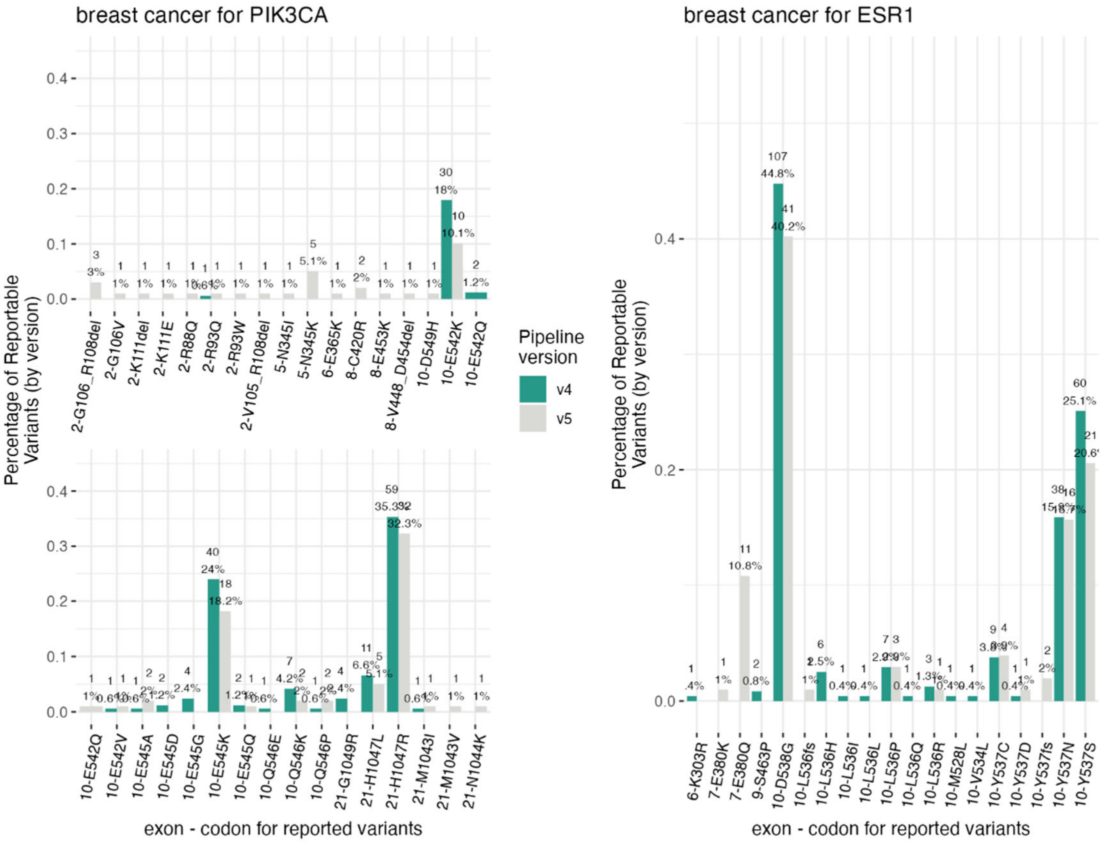

Figure S2. Relative mutation frequency in the lung cancer cohort for select genes.

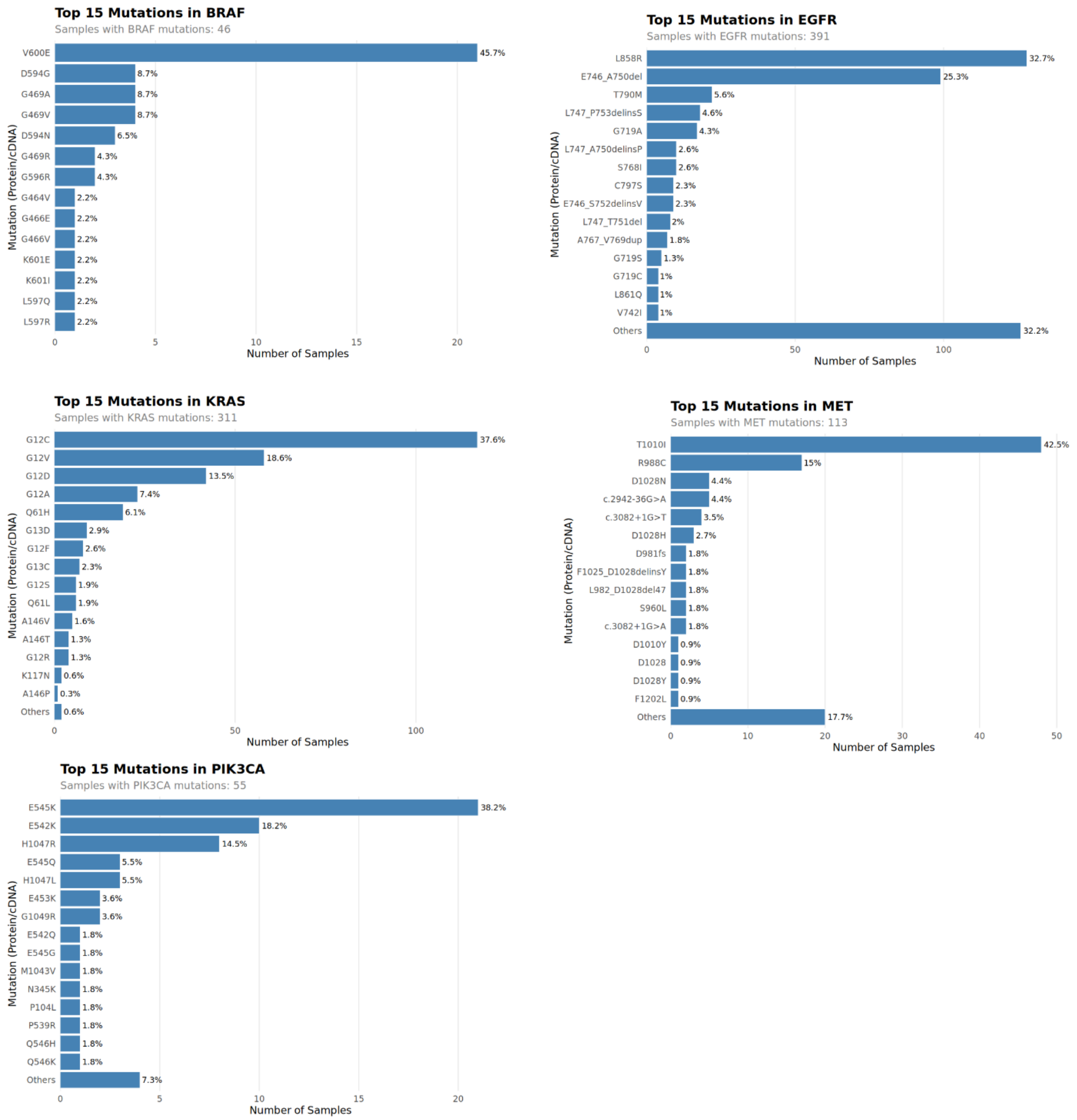

Figure S3. The model structure for the testing component of standard of care arm;

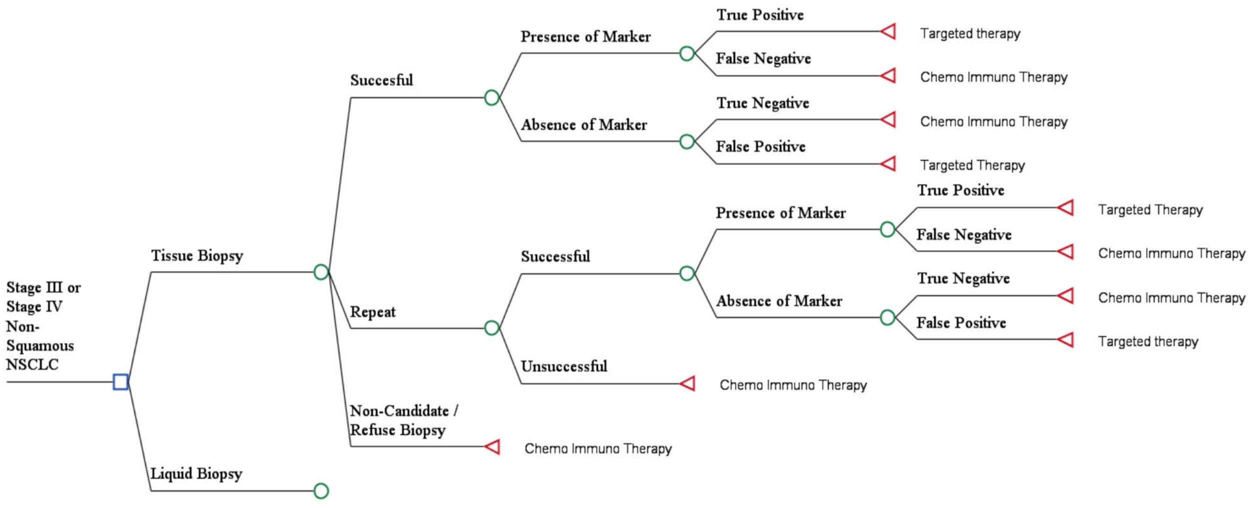

Figure S4. The model structure for the testing component of Follow It arm.

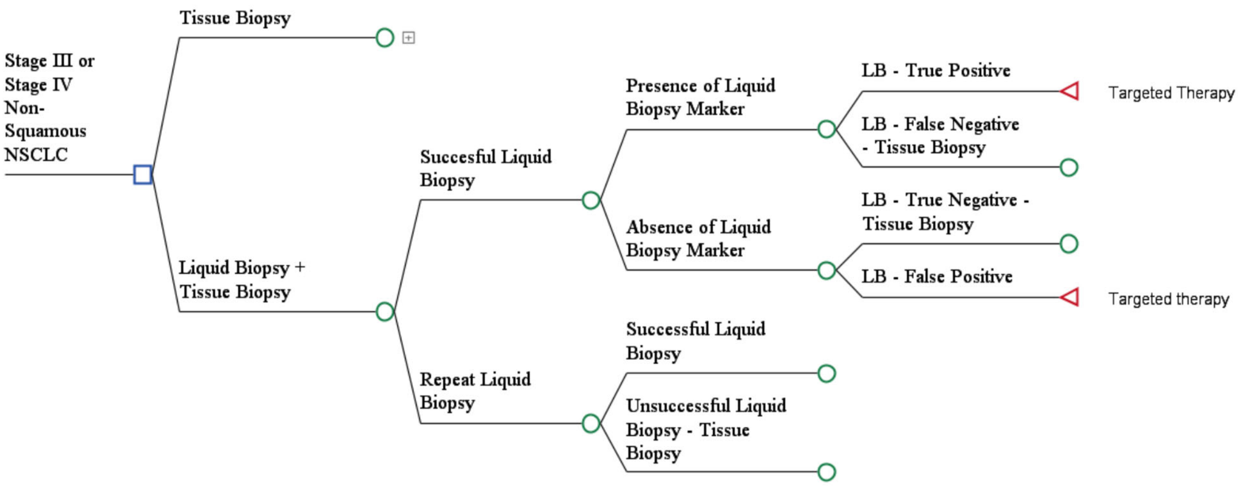

Figure S5. Markov models for patients receiving targeted therapy or chemotherapy.

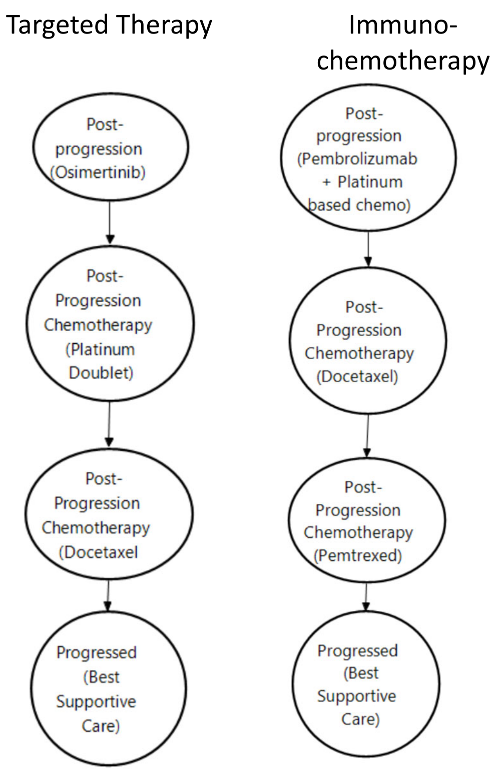

Supplementary Methods

This section describes details of the cost and health benefit analysis.

Survival and Progression

The progression free survival (PFS) and survival for Osimertinib are informed by Figures 2A and 3A in Mann, Andersohn[1]. PFS and overall survival for Pembrolizumab & platinum-based chemotherapy (carboplatin + pemetrexed) are informed by Gandhi, Rodriguez-Abreu[2]. The remaining clinical effective parameters for post first line treatment are shown in Table 6 below. The ratio of median time to progression reported in Oxnard, Thress[3] is used to derive a hazard ratio (2.85) for patients who are mutation negative compared with patients who are mutation positive treated with Osimertinib which is applied to progression free survival. The same hazard ratio is also applied to overall survival for patients who are mutation negative treated with Osimertinib.

Mortality rates are assumed to follow those of first line treatments, age-specific rates of all-cause mortality were obtained from Statistics Canada Life Tables.

Table S6. Clinical effectiveness parameters, post first-line treatment

Model Parameters	Median PFS in Months	Reference / notes
Platinum-based doublet chemotherapy	4.4 (4.2-5.6)	[4]
Docetaxel	3.8 (3.0-8.1)	[5]

Pemetrexed	4.2 (4.1–5.4)	[4]

Biopsy

The parameters that describe the implementation of tissue and liquid biopsy in the analysis are shown in Table 7 below.

Table S7. Biopsy parameters

Parameter	Value	Reference / notes
Sensitivity Tissue Biopsy	0.861 (0.759-0.981)	[6]assume an imperfect reference standard to calculate accuracy
Specificity Tissue Biopsy	0.934 (0.855-0.995)	“”
Proportion who cannot have liquid biopsy (i.e., refuse to have biopsy or noncandidate)	0	[6]
Proportion who cannot have tissue biopsy (i.e., refuse to have biopsy or noncandidate)	0.18 (95% CI: 0.1 to 0.38)	“”
Proportion of liquid biopsies that fail (i.e., test failure)	0.03 (0-0.08)	“”
Proportion of patients who receive repeat liquid biopsy after treatment failure	1	“”
Proportion of tissue biopsies that fail (i.e., test failure or inadequate tissue)	0.14 (95% CI: 0.13-0.15)	Up to 10–20% of biopsies are inadequate for molecular testing due to insufficient tissue or amplifiable DNA.[7]
Proportion of patients who receive repeat tissue biopsy after treatment failure	0.075 (0.05 to 0.1)	“”
Pneumothorax due to tissue biopsy	28% (0% to 61%)	[8,9]
% Pneumothorax cases that require chest drainage	30% (20% to 40%)	[6]

An important consideration in this analysis is the delay in receiving a tissue biopsy and its impact on patient outcomes. Clinical studies show that the necessity for re-biopsy can lead to increased risks and costs and be challenging from the patient perspective[10]. Moreover, delays on the patient pathway that are associated with tissue biopsy can lead to adverse patient outcomes[11] and are incorporated into the analysis (see below).

Delays

The impact of delays on the test and treatment pathway for lung cancer has been examined in the literature[11-14].

Table S8. Delay parameters.

Parameter	Value	Source
% Patients on tissue biopsy pathway that require hospitalization	16/101	[11]
Length of hospitalization	17 days (range 1 -30)	“”

Costs

The costs incorporated into this analysis were informed using the best available secondary sources, and are shown in Table 6 below.

Table S9: Costs

Parameter	28-day cycle	Notes	Source
Osimertinib	$8,466.92	80 mg one daily ($302.39), price 28-day cycle	[6] updated from 2018 prices
Platinum based doublet	$340.70	Pemetrexed: 500 mg/m^2 per cycle; Cisplatin: 75 mg/m^2	“”
Pemetrexed maintenance	$249.48	500 mg/m^2 per cycle	“”
Docetaxel	$98.52	75 mg/m^2 per cycle	“”
Pembrolizumab + Platinum-based chemotherapy (carboplatin + pemetrexed):			
Pembrolizumab	$12,040.54	$4,400 per 100mg vial • Cost per dose: $8800.00 • Cost per 28 days: $11,733 (2018 prices)	[15]
Carboplatin	$126.86	• $18.80 per 150 mg vial; • $56.39 per 450 mg vial; • Cost per dose (645 mg): $87.41; • Cost per 28 days: $116.55 (2012 prices)	“”
Pemetrexed	$4,072	When wastage is taken into consideration, the cost of a 28-day course is $3,968 (2012 prices)	“”
Unit Cost			
Tissue biopsy	$2,595	Average: commercial RT-PCR and in-house dPCR (updated from 2018 prices)	[6]
Pneumothorax:<br>Chest Drain<br>X-ray	 $487.45<br>$442.30		“”
One day in hospital due to treatment delays	$474.10		CIHI

Table S10: Drug administration and monitoring costs

Treatment type	Cost per 28-day cycle (SE)	Source
Osimertinib	$26.01 (3.31)	[6,16]
Platinum-based doublet	$232.07 (29.60)	“”
Pemetrexed maintenance	$86.27 (11.00)	“”
Docetaxel	$200.69 (25.60)	“”
Pembrolizumab	$26.01 (3.31)	Assume the same as Osimertinib

Table S11: General care & end of life costs

Disease Status	Cost per 28-day cycle	Source
Progression-free	$1055.18	[6,16]
Progressed-disease	$1248.46	“”
End of life	$14,990.89	[17]

Utilities

The health utility of patients as they transition through each of the health states described in the Markov models is given in Table 12.

Table S12: Health utilities

Parameter	Value	Source
Osimertinib	0.652	[6,18]
Platinum-based doublet	0.609 (0.409 to 0.809, assumed)	“”
Docetaxel	0.551 (20% variation, assumed	“”
Best supportive care	0.474 (20% variation, assumed)	“”
Disease progression	0.474 (20% assumed)	“”
Pneumothorax requiring pleural drainage	0.450 (0.33 to 0.58)	[19]
Pembrolizumab / pemetrexed	0.67 (0.47 to 0.87)	[20]

Budget impact analysis

The results of the budget impact analysis are shown in Table 13.

Table S13: Budget Impact Analysis

Year	Total	Treatment	End of Life	Biopsy	Delay	Drug Costs Targeted Therapy	Drug Costs Immuno Chemotherapy
1	-$74,282,100	-$71,304,455	-$2,515,209	$1,024,111	-$1,486,547	$91,706,187	-$163,916,911
2	-$93,916,784	-$91,209,594	-$2,252,490	$1,031,847	-$1,486,547	$130,086,205	-$223,239,239
3	-$97,902,007	-$95,538,161	-$1,919,072	$1,041,773	-$1,486,547	$147,788,523	-$246,100,056
4	-$88,700,170	-$86,500,280	-$1,762,742	$1,049,399	-$1,486,547	$155,921,944	-$246,292,368
5	-$84,458,159	-$82,336,961	-$1,688,816	$1,054,165	-$1,486,547	$159,650,328	-$246,372,561

References

1. Mann, H.; Andersohn, F.; Bodnar, C.; Mitsudomi, T.; Mok, T.S.K.; Yang, J.C.; Hoyle, C. Adjusted Indirect Comparison Using Propensity Score Matching of Osimertinib to Platinum-Based Doublet Chemotherapy in Patients with EGFRm T790M NSCLC Who Have Progressed after EGFR-TKI. *Clin Drug Investig* **2018**, *38*, 319-331, doi:10.1007/s40261-017-0611-3.
2. Gandhi, L.; Rodríguez-Abreu, D.; Gadgeel, S.; Esteban, E.; Felip, E.; De Angelis, F.; Domine, M.; Clingan, P.; Hochmair, M.J.; Powell, S.F.; et al. Pembrolizumab plus Chemotherapy in Metastatic Non-Small-Cell Lung Cancer. *N Engl J Med* **2018**, *378*, 2078-2092, doi:10.1056/NEJMoa1801005.
3. Oxnard, G.R.; Thress, K.S.; Alden, R.S.; Lawrance, R.; Paweletz, C.P.; Cantarini, M.; Yang, J.C.; Barrett, J.C.; Jänne, P.A. Association Between Plasma Genotyping and Outcomes of Treatment

With Osimertinib (AZD9291) in Advanced Non-Small-Cell Lung Cancer. *J Clin Oncol* **2016**, *34*, 3375-3382, doi:10.1200/jco.2016.66.7162.
4. Mok, T.S.; Wu, Y.L.; Ahn, M.J.; Garassino, M.C.; Kim, H.R.; Ramalingam, S.S.; Shepherd, F.A.; He, Y.; Akamatsu, H.; Theelen, W.S.; et al. Osimertinib or Platinum-Pemetrexed in EGFR T790M-Positive Lung Cancer. *N Engl J Med* **2017**, *376*, 629-640, doi:10.1056/NEJMoa1612674.
5. Horn, L.; Spigel, D.R.; Vokes, E.E.; Holgado, E.; Ready, N.; Steins, M.; Poddubskaya, E.; Borghaei, H.; Felip, E.; Paz-Ares, L.; et al. Nivolumab Versus Docetaxel in Previously Treated Patients With Advanced Non-Small-Cell Lung Cancer: Two-Year Outcomes From Two Randomized, Open-Label, Phase III Trials (CheckMate 017 and CheckMate 057). *J Clin Oncol* **2017**, *35*, 3924-3933, doi:10.1200/jco.2017.74.3062.
6. Cell-Free Circulating Tumour DNA Blood Testing to Detect EGFR T790M Mutation in People With Advanced Non-Small Cell Lung Cancer: A Health Technology Assessment. *Ont Health Technol Assess Ser* **2020**, *20*, 1-176.
7. Arcila, M.E.; Oxnard, G.R.; Nafa, K.; Riely, G.J.; Solomon, S.B.; Zakowski, M.F.; Kris, M.G.; Pao, W.; Miller, V.A.; Ladanyi, M. Rebiopsy of lung cancer patients with acquired resistance to EGFR inhibitors and enhanced detection of the T790M mutation using a locked nucleic acid-based assay. *Clin Cancer Res* **2011**, *17*, 1169-1180, doi:10.1158/1078-0432.Ccr-10-2277.
8. Ayyappan, A.P.; Souza, C.A.; Seely, J.; Peterson, R.; Dennie, C.; Matzinger, F. Ultrathin fine-needle aspiration biopsy of the lung with transfissural approach: does it increase the risk of pneumothorax? *AJR Am J Roentgenol* **2008**, *191*, 1725-1729, doi:10.2214/ajr.08.1110.
9. McLean, A.E.B.; Barnes, D.J.; Troy, L.K. Diagnosing Lung Cancer: The Complexities of Obtaining a Tissue Diagnosis in the Era of Minimally Invasive and Personalised Medicine. *J Clin Med* **2018**, *7*, doi:10.3390/jcm7070163.
10. Pennell, N.A.; Arcila, M.E.; Gandara, D.R.; West, H. Biomarker Testing for Patients With Advanced Non-Small Cell Lung Cancer: Real-World Issues and Tough Choices. *Am Soc Clin Oncol Educ Book* **2019**, *39*, 531-542, doi:10.1200/edbk_237863.
11. Blanc-Durand, F.; Florescu, M.; Tehfe, M.; Routy, B.; Alameddine, R.; Tran-Thanh, D.; Blais, N. Improvement of EGFR Testing over the Last Decade and Impact of Delaying TKI Initiation. *Curr Oncol* **2021**, *28*, 1045-1055, doi:10.3390/curroncol28020102.
12. Vinas, F.; Ben Hassen, I.; Jabot, L.; Monnet, I.; Chouaid, C. Delays for diagnosis and treatment of lung cancers: a systematic review. *Clin Respir J* **2016**, *10*, 267-271, doi:10.1111/crj.12217.
13. Chandra, S.; Mohan, A.; Guleria, R.; Singh, V.; Yadav, P. Delays during the diagnostic evaluation and treatment of lung cancer. *Asian Pac J Cancer Prev* **2009**, *10*, 453-456.
14. Gomez, D.R.; Liao, K.P.; Swisher, S.G.; Blumenschein, G.R.; Erasmus, J.J., Jr.; Buchholz, T.A.; Giordano, S.H.; Smith, B.D. Time to treatment as a quality metric in lung cancer: Staging studies, time to treatment, and patient survival. *Radiother Oncol* **2015**, *115*, 257-263, doi:10.1016/j.radonc.2015.04.010.
15. Pan-Canadian Oncology Drug Review. Pemetrexed (alimta) for non-squamous non-small cell lung cancer. . *Toronto (ON): Pan-Canadian Oncology Drug Review* **2013**.
16. Goeree, R.; Villeneuve, J.; Goeree, J.; Penrod, J.R.; Orsini, L.; Tahami Monfared, A.A. Economic evaluation of nivolumab for the treatment of second-line advanced squamous NSCLC in Canada: a comparison of modeling approaches to estimate and extrapolate survival outcomes. *J Med Econ* **2016**, *19*, 630-644, doi:10.3111/13696998.2016.1151432.
17. Cheung, M.C.; Earle, C.C.; Rangrej, J.; Ho, T.H.; Liu, N.; Barbera, L.; Saskin, R.; Porter, J.; Seung, S.J.; Mittmann, N. Impact of aggressive management and palliative care on cancer costs in the final month of life. *Cancer* **2015**, *121*, 3307-3315, doi:10.1002/cncr.29485.
18. Nafees, B.; Stafford, M.; Gavriel, S.; Bhalla, S.; Watkins, J. Health state utilities for non small cell lung cancer. *Health Qual Life Outcomes* **2008**, *6*, 84, doi:10.1186/1477-7525-6-84.

19. Morimoto, T.; Fukui, T.; Koyama, H.; Noguchi, Y.; Shimbo, T. Optimal strategy for the first episode of primary spontaneous pneumothorax in young men. A decision analysis. *J Gen Intern Med* **2002**, *17*, 193-202, doi:10.1046/j.1525-1497.2002.10636.x.
20. Weng, X.; Luo, S.; Lin, S.; Zhong, L.; Li, M.; Xin, R.; Huang, P.; Xu, X. Cost-Utility Analysis of Pembrolizumab Versus Chemotherapy as First-Line Treatment for Metastatic Non-Small Cell Lung Cancer With Different PD-L1 Expression Levels. *Oncol Res* **2020**, *28*, 117-125, doi:10.3727/096504019x15707883083132.
